# Estimating Human Wrist Stiffness during a Tooling Task

**DOI:** 10.3390/s20113260

**Published:** 2020-06-08

**Authors:** Gia-Hoang Phan, Clint Hansen, Paolo Tommasino, Aamani Budhota, Dhanya Menoth Mohan, Asif Hussain, Etienne Burdet, Domenico Campolo

**Affiliations:** 1 Robotics Research Center, School of Mechanical and Aerospace Engineering, Nanyang Technological University, Singapore 639798, Singapore; GIAHOANG001@e.ntu.edu.sg (G.-H.P.); PAOLO001@e.ntu.edu.sg (P.T.); BUDH0002@e.ntu.edu.sg (A.B.); dhanya.mohan@ku.ac.ae (D.M.M.); ahussain@articares.com (A.H.); e.burdet@imperial.ac.uk (E.B.); D.CAMPOLO@ntu.edu.sg (D.C.); 2 Neurogeriatrics Kiel, Department of Neurology, University Hospital of Kiel, 24105 Kiel, Germany; 3 Department of Bioengineering, Imperial College, London SW7 2BY, UK

**Keywords:** instrumented tool, tool-workpiece interaction, human joint stiffness

## Abstract

In this work, we propose a practical approach to estimate human joint stiffness during tooling tasks for the purpose of programming a robot by demonstration. More specifically, we estimate the stiffness along the wrist radial-ulnar deviation while a human operator performs flexion-extension movements during a polishing task. The joint stiffness information allows to transfer skills from expert human operators to industrial robots. A typical hand-held, abrasive tool used by humans during finishing tasks was instrumented at the handle (through which both robots and humans are attached to the tool) to assess the 3D force/torque interactions between operator and tool during finishing task, as well as the 3D kinematics of the tool itself. Building upon stochastic methods for human arm impedance estimation, the novelty of our approach is that we rely on the natural variability taking place during the multi-passes task itself to estimate (neuro-)mechanical impedance during motion. Our apparatus (hand-held, finishing tool instrumented with motion capture and multi-axis force/torque sensors) and algorithms (for filtering and impedance estimation) were first tested on an impedance-controlled industrial robot carrying out the finishing task of interest, where the impedance could be pre-programmed. We were able to accurately estimate impedance in this case. The same apparatus and algorithms were then applied to the same task performed by a human operators. The stiffness values of the human operator, at different force level, correlated positively with the muscular activity, measured during the same task.

## 1. Introduction

From a robotic perspective, one of the most interesting features of humans is their proficiency to operate a variety of tools in order to interact with and adapt to an often unpredictable environment. For example, artisans can skilfully use scalpels, hammers and grinders to carve, bend or polish different types of materials and geometries. While humans can cope with tooling tasks seemingly effortless and can flexibly adapt their motor strategies to different mechanical and geometrical constraints [1,2,3], for conventional industrial robots these type of tasks remain challenging.

Tooling an object with a robot is challenging for several reasons: (i) the mechanical properties of the tooled object are usually unknown; (ii) when tooling an object, its surface may deform or wear out and therefore the robot controller can no longer rely on the original CAD model and must continuously adapt to changes of the surface geometry; (iii) friction and surface irregularities may result in large forces and to instability, especially for high gain feedback controllers typically adopted in industrial manipulators [4,5]. Conventional approaches based on model-development, robot programming and fine-tuning (with possibly several iterations of these steps) are often not suitable to recent high-mix low-volume manufacturing trends. For example, the process of programming a robot with a teaching pendant (as industrial practice) is exceedingly time-consuming, especially given the relatively short life-time of modern products.

One possibility is to move beyond traditional robot programming and make use of teaching by demonstration [6]. This is not only a way to speed up the process but it also allows to capture tacit knowledge of an experienced operator in a more natural way [7]. The tacit knowledge we aim at capturing in this work is the level of biomechanical stiffness displayed by the operator during contact tasks. As explained below, the novelty of our approach is in the exploitation of the natural variability of the task as a means to evaluate stiffness via regressive methods, as opposed to using ad-hoc robotic devices to generate disturbances, e.g., [1,2,3,8].

Hybrid position/force control is often implemented for tooling tasks such as polishing whereby a position controller is used to align the tool with the relevant geometric features of the surface under tooling while a force controller applies desired forces in specific directions with respect to the surface [9]. Hybrid position/force control approaches, however, may fail in presence of unstable contact tasks such as drilling and carving and human operators are still required to carry out these so-called finesse finishing tasks.

What mechanisms allow humans to perform such tasks? Several behavioural studies have shown that the success of humans to cope with unstable and unpredictable environments resides in their capability to adapt the endpoint mechanical impedance to the environmental conditions [2,10,11,12]. These studies have led to novel bioinspired controllers mimicking the impedance characteristics of human operators performing contact tasks [4,5,13,14,15,16].

In this paper we focus on learning by demonstration, in particular on the estimation of human wrist stiffness during tooling tasks, specifically in the case of hand-held tools. When a human operator performs a tooling task, typically the entire posture of arm/torso is involved, accounting for more than a dozen degrees of freedom (dof). However, within a learning by demonstration framework, one is ultimately interested in mapping a human demonstration onto an industrial robot, typically with much fewer degrees of freedom. Not to be robot-specific, in this paper will focus on tasks such as polishing of relatively short and straight edges, which can be accomplished by most robots, with at least 2 dof. This gives rise to what in imitation learning is known as correspondence problem [17], i.e., mapping motions from a demonstrator to a learner when these two agents are kinematically dissimilar. In addition to this problem, different demonstrators might have different ways to perform the same task, with varying emphasis on motion of distal and proximal joints depending, for example, on their specific stature, age and musculature (hand-held power tools can be quite heavy). To guide the operator, we make use of an arm-rest, so that the task will be accomplished mainly through the operator’s wrist motions.

Mechanical impedance is a mathematical operator which predicts the force generated by a system in response to an imposed motion [18]. Impedance operators are typically defined for linear systems but can also be generalized to nonlinear systems once the system is linearized and under the assumption of ‘small perturbations’. When an external perturbation displaces the hand, a restoring force brings it back to the initial position [19,20] or to the undisturbed trajectory [21,22,23]. It is also possible to estimate stiffness from a single movement [24] however we chose a dynamic tooling-task and apply regression methods.

By linearizing the system behaviour around a given equilibrium point or trajectory, the impedance can be decomposed into: stiffness, the resistance to a change in position; damping, the resistance to a change in velocity; and inertia, the resistance to a change in acceleration. For multiple-joint systems, such as a human arm, this process must be generalized to account for geometric factors, and the multi-dimensionality of interaction dynamics and kinematics [25,26,27,28,29]. In humans, while inertia due to the masses of bones and muscles cannot be controlled (for any given posture), stiffness and damping are due to the viscoelastic behaviour of muscles, tendons and ligaments and to reflexes, influenced by the motor commands [30].

In studies involving humans, the source of perturbation is usually a robotic manipulator whose end-effector is directly connected to the anatomical part of interest, such as the subject’s hand [8,19,20,31]. Perturbations can be applied around an equilibrium configuration in static posture or during the undisturbed trajectory during movement. Several techniques have been proposed for the estimation of the hand impedance during movements. In this case, one of the main challenge, is the correct estimation of the unperturbed hand trajectory that is used to linearize the system dynamic [23].

A first approach to multi-joint hand impedance identification was based on displacement or force perturbations along different directions of the transverse plane [19,20]. By recording the restoring force and the hand kinematic following the perturbations, linear least square method was commonly employed to identify the parameters of the hand mechanical impedance. Due to noise in the force measurement and error in the movement prediction [23], such an approach usually requires several experimental trials (each for every direction of perturbations).

An alternative approach was proposed by Perreault et al. whereby a stochastic force [32] or position [33] perturbation (rather than a single displacement [23] or force pulse [22]) was used. Potential advantages of this method include: (i) the entire stiffness field could in principle be swept during a single or fewer trials (ii) due to the stochastic nature, this kind of perturbation is less likely to evoke voluntary responses and hence alter the quality of the estimation. While the stochastic perturbation method was initially developed based on the assumption that the system of the arm controlled by the nervous system is a linear time invariant system, thus restricting its use to estimation of impedance in static postures, recent works have developed extensions to estimation in movements e.g., [34].

To our knowledge, all methods to estimate impedance in the literature were using a robot or a dedicated computer-controlled module [35,36] to disturb movement or force, which restricts these methods to laboratory settings. However, these settings have different dynamics than the ecological scenarios we would be interested in investigating in order to understand how humans control movements and interactions in real-life situations. Here, we thus propose to rely on disturbances which naturally arise during tooling tasks [37]. Building upon stochastic perturbation methods to assess impedance, we will use perturbations caused by the interaction of a tool with the environment in order to identify the mechanical impedance in real-life scenarios.

**Figure 1:**
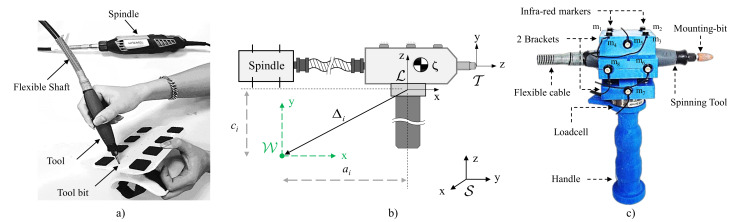
(**a**) Polishing tool’s description. Example of finishing task with a hand-held tool. (**b**) Schematic and naming conventions for the instrumented tool shown in (**c**).

The task of interest in this paper is polishing, a multi-pass process, typically performed by human operators via hand-held power-tools as illustrated in Figure 1. For one of such hand-held power-tools, we designed a custom handle which allows to capture (via multi-axis force/torque sensors) the 3D interaction dynamics between the tool and the operator. To capture the pose of the tool in 3D space, we used optical motion tracking with trackers affixed to the power-tool. The principle of this hand-held power-tool is described in [38]. The activity level of forearm/wrist muscles deemed relevant to the task and was consequently also monitored using surface electromyography (EMG).

The method for extracting impedance values during contact is first tested with an impedance-controlled industrial robot, deployed to perform a polishing task with a specific value of pre-set impedance. The same polishing task is then tested with a human operator using the power-tool. Data from this experiment are analysed and the impedance values are shown to positively correlate with the muscle activity of the forearm. In the following, Section 2 describes the method of joint stiffness assessment and its validation on an industrial impedance-controlled robot. Section 3 describes the protocols and experiments conducted on a human subject, data analysis and results.

## 2. Setup, Methods and Robotic Validation

We consider tooling tasks which involve non-negligible contact forces, such as grinding or polishing. More specifically, we assume that the tooling operation is conducted by means of a power hand-held tool rigidly attached to distal end of a wrist joint. Our technique to estimate stiffness using this tool will be tested and validated on a robotic wrist in this section. As the goal is to estimate the stiffness of a worker’s wrist when holding this tool, the coordinates definition will follow the anatomy of a human wrist.

### 2.1. Instrumented Tool: 3D Kinematics, Force and Torque Estimation

To measure 3D kinematics of the rigid tool, the tool was equipped with a redundant number of optical markers ensuring that at least three markers are always visible during operation. We consider a hand-held power tools operated with a single handle so that the interaction dynamics can be estimated using a single load cell capable of sensing 3D forces and 3D torques placed between the handle and the tool. For power tools, the spindle is usually the most heavy component and for some hand-held tools such as the Dremel 4000, it can be decoupled from the tool-bit via a flexible shaft, so that the user does not need to withstand the weight of the spindle during manual operations, as shown in Figure 1a).

In general, data relative to tool pose (3D positions and 3D orientations of a rigid body) and to interaction forces and torques [39] are acquired with respect to different reference frames, depending on the type of sensors (optical motion trackers and loadcells) and their placement. With reference to Figure 1b, for human/robot joint impedance analysis we shall refer all measurements to an anatomically relevant frame {W}, rigidly attached to the human/robot forearm and centred in the wrist. In this paper, we shall consider a simplified kinematic model of the human wrist, which we assume to be a spherical joint with 3 degrees of freedom (dof) expressed as a vector $q={\left[q^{RU}\;q^{FE}\;q^{PS}\right]}^{T}$, with the joint angles $q^{RU}$, $q^{FE}$, and $q^{PS}$ corresponding to radio ulnar-deviation (RU), flexion-extension (FE), and pronation-supination (PS), respectively. Despite this simplification of assuming a spherical joint, we shall still follow an anatomically correct sequence of rotations for the wrist joints (as in [40]) whereby PS is the most proximal joint and RU is the most distal. Defining the ‘zero’ configuration ($q={\left[0\;0\;0\right]}^{T}$) as the anatomically neutral position of the hand (i.e., the hand is aligned with the forearm, see [40] for details) the joint angles vector q can be derived from a 3×3 rotation matrix *R*, defined as the rotation of hand from its the neutral position. Following [40], the joint angles can be derived from *R* as:

(1) $$q=\left[\begin{array}{c} q^{RU}\\ q^{FE}\\ q^{PS} \end{array}\right]=\left[\begin{array}{c} \mathrm{atan}2(R_{1,3},R_{1,1})\\ \arcsin(-R_{1,2})\\ \mathrm{atan}2(-R_{3,2},R_{2,2}) \end{array}\right]$$

where $R_{i,j}$ represents the entry at the *i*-th row and *j*-th column of the matrix *R*.

**Figure 2:**
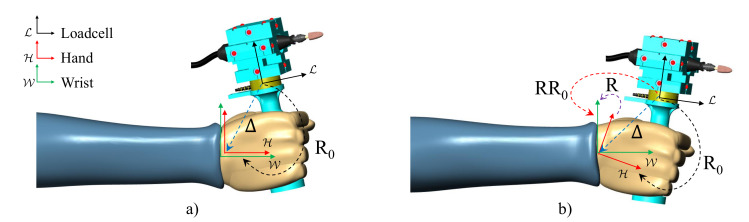
Neutral configuration of the hand. (**a**) Hand in neutral position; (**b**) Hand movement expressed by rotation R.

The 3D force ($F^{L}$) and torque ($T^{L}$) measurements are directly provided by the loadcell in coordinates relative to its intrinsic loadcell frame {L} and conveniently merged into a 6D wrench vector $W^{L}:={\left[F^{L}\;T^{L}\right]}^{T}$, accounts for the interaction between the tool-bit and the work-piece, but is corrupted by the effects of the flexible shaft and of the gravity on the tool. In order to determine torques in the wrist joint, the wrench $W^{L}$ needs to be transformed into wrist coordinates $W^{W}$. Taking into account the constant rotation $R_{0}$ and displacement $\Delta^{L}$ between loadcell and hand (from loadcell to hand coordinates), the hand rotation *R* (from hand to wrist coordinates, as shown in Figure 2), the wrench transformation can be computed as [9]

(2) $$W^{W}=\left[\begin{array}{c} F^{W}\\ T^{W} \end{array}\right]=\left[\begin{array}{cc} R\;R_{0} & 0\\ R\;R_{0}\left[\Delta^{L}\times \right] & R\;R_{0} \end{array}\right]W^{L}$$

where $\left[\Delta^{L}\times \right]$ is the anti-symmetric matrix associated (To any vector $a\in R^{3}$, can associate a skew-symmetric matrix [a×] defined such that [a×]b=a×b, for any vector $b\in R^{3}$[9].) with the vector $\Delta^{L}$.

Finally, the anatomical joint torques can then be extracted from the wrench as

(3) $$\left[\begin{array}{c} T^{RU}\\ T^{FE}\\ T^{PS} \end{array}\right]:=T^{W}=\left[\begin{array}{cccccc} 0 & 0 & 0 & 1 & 0 & 0\\ 0 & 0 & 0 & 0 & 1 & 0\\ 0 & 0 & 0 & 0 & 0 & 1 \end{array}\right]W^{W}$$

### 2.2. Compliant Robots: Impedance-Controlled Joints

In contrast to conventional robotic manipulators that are typically stiff and position-controlled, modern service and industrial robots embed high-bandwidth current servos and/or joint-torque sensors allowing force/impedance control strategies. Several impedance/force control approaches have been proposed in the last two decades and the reader is referred to [41] for the literature on this subject. In this work, we shall refer to the impedance-control schemes available for commercial manipulators such as the KUKA lightweight robot [42].

**Figure 3:**
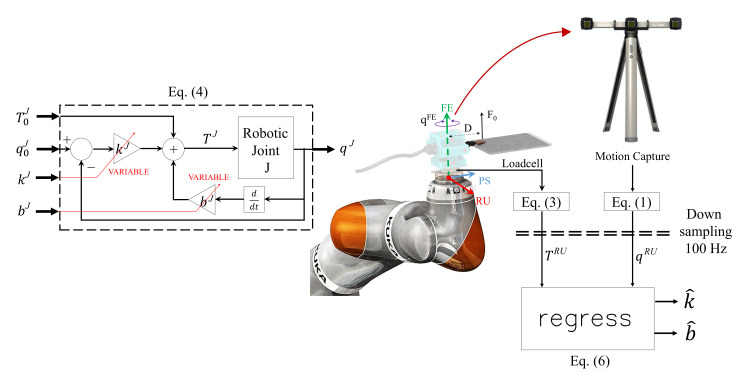
Method to estimate a robot stiffness and damping during polishing while moving sinusoidally. The top left corner shows a diagram of a torque-controlled robotic joint.

The impedance-controlled robot used in this work was programmed in operative (or Cartesian) mode. In this modality, a Cartesian frame of reference is defined at the wrist of the robot, as shown in Figure 3. The six degrees of freedom (dof) of this Cartesian frame formed of 3D translations and 3D rotations will hereafter be referred to as ‘joints’. Furthermore, as the rotations of the Cartesian frame will be used to mimic the wrist rotations of the human operator, we shall denote these axes as radial-ulnar deviation (RU), flexion-extension (FE), and pronation-supination (PS), as indicated in Figure 3.

Using the subscript *J* to denote different joints (i.e., J≡RU,FE,orPS), we shall consider an impedance-control mode with overlaid (i.e., superimposed) torques. Specifically, we shall assume that each robotic joint *J* is controlled according to

(4) $$T^{J}=T_{0}^{J}+k^{J}\left(q^{J}-q_{0}^{J}\right)+b^{J}{\dot{q}}^{J}\;J\equiv RU,FE,\mathrm{or}\;PS$$

where $k^{J}$ is the joint stiffness and $b^{J}$ joint damping, $q_{0}^{J}$ is the desired trajectory, $q^{J}$ is the actual trajectory, ${\dot{q}}^{J}$ denotes the velocity, $T_{0}^{J}$ is the overlaid torque and $T^{J}$ is the commanded joint torque. This scheme, also shown in Figure 3, assumes that the robotic platform is force-controlled [43], i.e., forces at each joints are commanded based on the readings from encoders. The joint position $q^{J}$ is measured via encoders and a control torque $T^{J}$ is computed via Equation (4) and then commanded to the joint actuator. To ensure stability, the control torque $T^{J}$ should be updated (1 kHz) to respond to changes in joint angle $q^{J}$, e.g., fast vibrations. Parameters such as $T_{0}^{J}$, $q_{0}^{J}$, $k^{J}$, $b^{J}$ are meant to be input commands modulated at a much slower rate, e.g., comparable to human motions.

Also illustrated in Figure 3, the interaction torque of the instrumented tool is measured at 1 kHz by an ATI mini 40 loadcell and its position at 100 Hz by motion capture (VZ4000, Poenix Technologies Inc.), spatial resolution 0.015 mm at 1.2 m distance. The anatomical joint torque and angle can then be determined using Equations (1) and (3). The rate of torque is downsampled to 100 Hz to match the frequency of the motion capture. The digital downsampling was accomplished using the Matlab function downsample. We reduced the sampling frequency of 1000 Hz to 100 Hz by keeping the first sample and then every 10th sample after the first. Finally, stiffness and damping of the KUKA robot are determined from the joint torque and angle, using the Equation (6) which will be presented later in this section. Importantly, the stiffness $k^{J}$ and damping $b^{J}$ are assumed to be constant during a trial. This is certainly true for the robot experiment below but it is important to note that impedance can change during movement of a human operator.

### 2.3. Robotic Impedance Estimation during Tooling Tasks

Although robots generally provide access to joint angles $q^{J}$ and commanded torques $T^{J}$, here we shall solely rely on measurements derived from motion capture systems and external loadcells, via Equations (1) and (3), as these will be the only source of information for experiments performed by humans. Furthermore, in this paper, we focus on the RU-axis so on the measurements $T_{0}^{RU}$, $k^{RU}$, and $q_{0}^{RU}$ as per Equation (4). We tested the estimation process illustrated in Figure 3 on the KUKA lightweight robot, which was controlled in impedance to perform a tooling task kinematically similar to the human task described in next section. This task mainly involves a periodic, slow and self-paced oscillating motion about the FE-axis while maintaining a constant torque about the RU-axis. We focus here on estimating impedance only along the RU-axis.

To this end, the robot was programmed to be rotationally compliant (with rotational stiffness 10 Nm/rad and damping 1.9 Nm s/rad) along the FE- and RU-axis, as shown in Figure 3, while maintaining maximum stiffness along the remaining dof (i.e., 300 Nm/rad rotational stiffness along the PS-axis and 5000 N/m translational stiffness in all directions). With reference to Equation (4), the following desired trajectories $q_{0}^{J}$ (J=FE,RU) and overlaid torques $T_{0}^{J}$ (J=FE,RU) where pre-programmed for the FE- and RU-axes:

(5) $$\mathrm{FE}-\mathrm{axis}\left\{\begin{array}{ccc} T_{0}^{FE} & = & 0\;Nm\\ q_{0}^{FE} & = & \frac{\pi }{6}\sin\left(3t\right)\;rad\\ k^{FE} & = & 10\;Nm/rad\\ b^{FE} & = & 1.9\;Nms/rad \end{array}\right.\;\mathrm{RU}-\mathrm{axis}\left\{\begin{array}{ccc} T_{0}^{RU} & = & 0.5\;Nm\\ q_{0}^{RU} & = & 0\;rad\\ k^{RU} & = & 10\;Nm/rad\\ b^{RU} & = & 1.9\;Nms/rad \end{array}\right.$$

The sinusoidal motion imposed to $q_{0}^{FE}$ in Equation (5) takes approximately 2 s for a whole period. In order to obtain 10 repetitions for statistical analysis, we performed the experiments for approximately 20 s, during which joint positions $q_{i}^{FE}$, $q_{i}^{RU}$ where sampled from Equation (1) at 100 Hz and joint torques $T_{i}^{FE}$, $T_{i}^{RU}$ where computed from Equation (3).

Considering a sequence measurements $q_{i}^{RU}$ and $T_{i}^{RU}$, at each time sample $t_{i}$, Equation (4) can be rewritten as:

(6) $$\left[\begin{array}{c} T_{i}^{RU} \end{array}\right]=T_{avg}^{RU}+\left[\begin{array}{cc} q_{i}^{RU} & {\dot{q}}_{i}^{RU} \end{array}\right]\left[\begin{array}{c} \hat{k}\\ \hat{b} \end{array}\right]$$

where to regress the impedance (formed of stiffness $\hat{k}$ and damping $\hat{b}$ ), or as

(7) $$\left[\begin{array}{c} T_{i}^{RU} \end{array}\right]=T_{avg}^{RU}+\left[\begin{array}{c} q_{i}^{RU} \end{array}\right]{\hat{k}}^{\prime }$$

where to estimate only stiffness ${\hat{k}}^{\prime }$. In these equations the scalar $T_{avg}^{RU}$ represents the average torque $T_{i}^{RU}$ over one period of time, which accounts for both the constant torque $T_{0}^{RU}$ and the factor $k^{RU}q_{0}^{RU}$. The terms in square brackets represent matrices of measurements.

**Figure 4:**
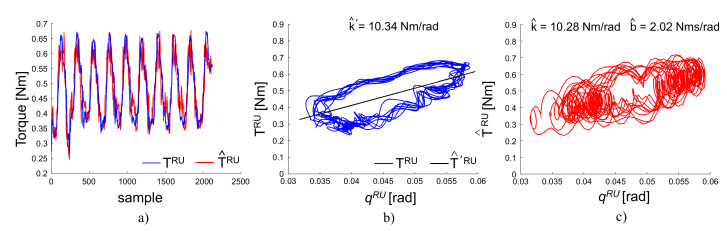
Result of impedance identification during the validation experiment with a robot. (**a**) Measured torque $T^{RU}$ vs. estimated torque ${\hat{T}}^{RU}$. (**b**) Estimated torque ${\hat{T}}^{\prime RU}$ using an elastic model. (**c**) Estimated torque ${\hat{T}}^{RU}$ with visco-elastic model.

To assess the goodness of regression, the measured torques were compared with the estimated viscoelastic ${\hat{T}}^{RU}$ and purely-elastic ${\hat{T}}^{\prime RU}$ torques, as predicted from Equations (6) and (7), respectively, defined as follows:

(8) $${\hat{T}}^{RU}=T_{avg}^{RU}+\hat{k}\;q^{RU}+\hat{b}\;{\dot{q}}^{RU}$$

(9) $${\hat{T}}^{\prime RU}=T_{avg}^{RU}+{\hat{k}}^{\prime }q^{RU}$$

where $q^{RU}$ and ${\dot{q}}^{RU}$ are the actually measured kinematics. $T_{avg}^{RU}$, $\hat{k}$, $\hat{b}$ and ${\hat{k}}^{{}^{\prime }}$ are regressed from Equations (6) and (7). As reported in Figure 4, when fitting only with an elastic model Equation (7), the regression returned a value of ${\hat{k}}^{\prime }=10.34$ Nm/rad. When fitting with the viscoelastic model in Equation (6), the regression returned a stiffness $\hat{k}=10.28$ Nm/rad and a damping $\hat{b}=2.02$ Nm.s/rad. In both cases, the stiffness and impedance estimates are accurate within 2% with respect to the pre-programmed k=10 Nm/rad and b=1.9 Nm.s/rad, as in Equation (5) for the RU-axis.

It should be noted that the same data acquisition system (in particular the same instrumented tool in Figure 1), algorithm and also a similar protocol as the one pre-programmed for the robot will be used to perform experiments with the human operator, so the same accuracy is expected in the estimation of impedance.

## 3. Estimation of Human Operators’ Joint Stiffness During Tooling Tasks

In this section we use the instrumented tool to estimate wrist kinematics and contact forces during a tooling task performed by a human operator. In addition, recording of muscle activity will enable us to study whether the identified impedance has properties expected from muscle physiology.

### 3.1. Experiments and Setup

**Figure 5:**
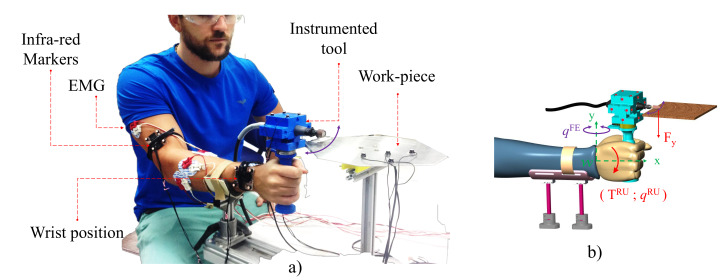
Human operator performing a polishing task. (**a**) Actual experimental setup. (**b**) Naming conventions. Please see online content for a video representation of the task including the visual feedback.

As shown in Figure 5, a the human operator was seated in front of the workpiece (the metal square plate used for the robot experiment) while grasping the tool’s handle with their right hand. The operator’s forearm was supported against gravity to reduce fatigue and was strapped to the support frame to confine the task to the wrist. The forearm height was adjusted to ensure that the tool’s tip was in contact with the workpiece at the beginning of the experiment. This initial configuration was set as neutral reference configuration $q^{PS}=q^{FE}=q^{RU}\equiv 0$ rad.

The operator was instructed to move the tool periodically left-and-right across the horizontal workpiece surface shown in Figure 5b at self-paced speed while keeping a desired interaction force $F_{y}>0$. No instruction was given regarding the speed and/or the range of horizontal movement. The tool tip rotated with a constant angular velocity of 15,000 rpm during the entire duration of the experiment.

**Figure 6:**
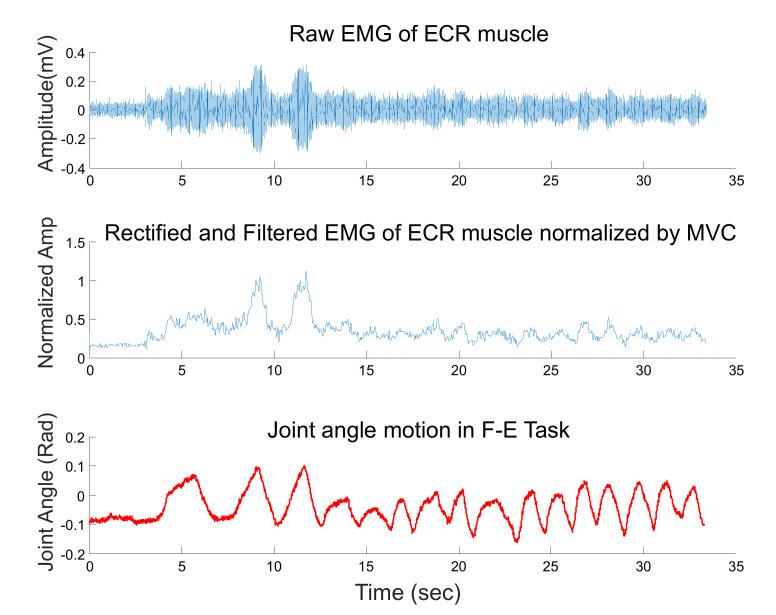
Steps of EMG pre-processing illustrated on for an ECR muscle of the operator.

We assume that the horizontal movement is mainly due to wrist flexion-extension and that the operator plans a constant reference trajectory $q_{0}^{RU}\left(t\right)=0$ rad for the RUD joint, therefore focus on the estimation of RUD wrist stiffness. During the experiment, surface electromyography (EMG) was used to monitor the activation of muscles representing the control of the wrist RUD rotation: extensor carpi radialis longus (ECR), extensor carpi ulnaris (ECU), flexor carpi radialis (FCR), and the flexor carpi ulnaris FCU [44]. EMG activity was collected with the BIOPAC MP150 Data Acquisition and Analysis System at 1 kHz. The raw EMG signal was pre-amplified with a gain value of G=1000 and band-pass filtered between 10–500 Hz. This signal was rectified, low-pass filtered (using a 4th order Butterworth filter with 20 Hz cut-off frequency [45]) to yield the EMG envelope, which was normalized with respect to the maximum activity displayed by the muscle during a maximum voluntary contraction (MVC) task performed before starting with the experiment (Figure 6). The mean normalised envelope was used as a measure of muscular effort and its contribution to the task.

### 3.2. Impedance and Muscle Effort Dependence on Force

One healthy operator (male, 29 years old, right-handed) conducted the experiment. A trial consisted of 10 forth and back rotational extension-flexion movements. Four conditions were tested corresponding to increasing levels of desired contact force:3N, 5N, 7N, 9N. For each condition, the operator was asked to perform 12 trials consecutively. To enable the subject to apply a nearly constant level of force on the plate during movement, visual feedback was given to the subject consisting of a bar displaying the error between the applied force and the desired level of contact force. The subject was instructed to keep the error as small as possible for the whole duration of the experiment.

**Figure 7:**
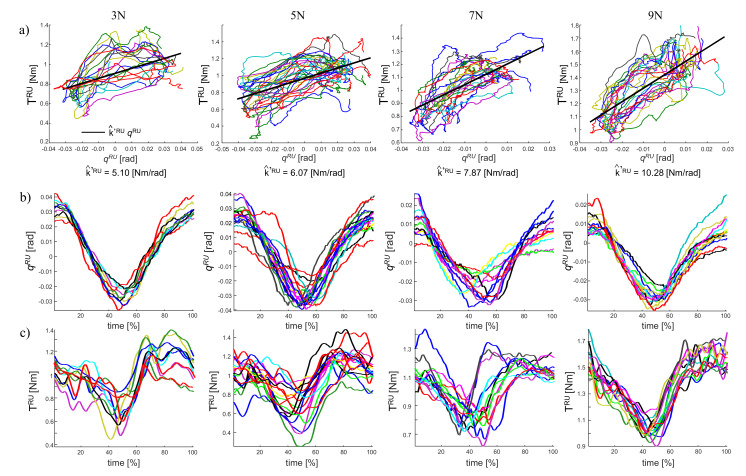
Results of the flexion-extension task. (**a**) The stiffness estimation is given from Equation (7). (**b**) The measured angle given from the motion capture. (**c**) The measured torque by the instrumented tool.

**Figure 8:**
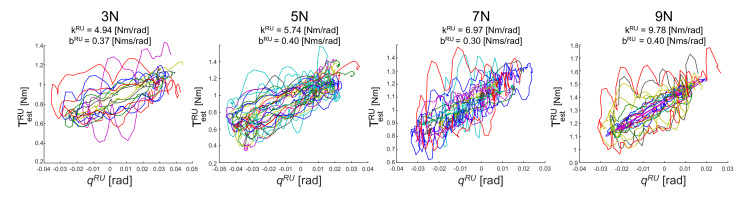
Estimation of wrist visco-elasticity during wrist-flexion-extension task.

**Figure 9:**
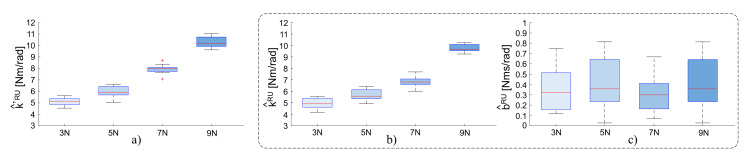
Results of the stiffness estimation for the radio-ulnar deviation. Stiffness distribution for four levels of applied forces: 3N, 5N, 7N, and 9N, respectively. (**a**) Estimated scalar stiffness ${\hat{k}}^{{}^{\prime }RU}$ from Equation (7). (**b**,**c**) Estimated scalar stiffness ${\hat{k}}^{RU}$ and damping ${\hat{b}}^{RU}$ from Equation (6).

Figure 7 shows the result for the flexion-extension task experiment, the estimated stiffness ${\hat{k}}^{{}^{\prime }RU}$ of the subject wrist from Equation (7) for the applied force of 3N, 5N, 7N, 9N is: 5.10 Nm/rad, 6.07 Nm/rad, 7.87 Nm/rad, and 10.28 Nm/rad, respectively. Using the Equation (6) presented above, the estimated torque from the estimated stiffness and damping is shown in Figure 8. As shown in Figure 9, the wrist stiffness of the subject increases in a roughly linear manner with the applied force for both methods of regression.

**Figure 10:**
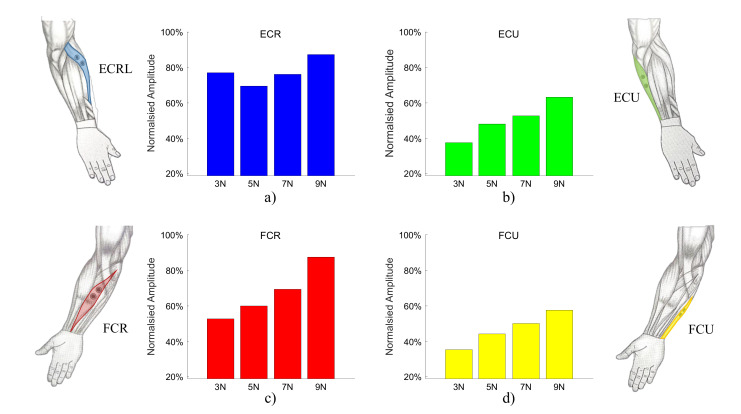
Muscular effort increases with the applied force for the (**a**) ECRL: Extensor Carpi Radials Longus, (**b**) ECU: Extensor Carpi Ulnaris, (**c**) FCR: Flexor Carpi Radialis, (**d**) FCU: Flexor Carpi Ulnaris. Values are shown as percentage of the maximum voluntary contraction (MVC).

In Figure 10 it can be observed that the mean muscle activity increases with the desired force level to be maintained, in a similar way as stiffness, as can be expected from the muscle physiology [30,46]. Among the wrist extensors and flexors, the ECR and FCR that are responsible for radial deviation show relatively higher activity as these muscles are used to hold the tool against the workpiece.

## 4. Conclusions and Discussion

In this work, we propose an instrumented tool and a regressive algorithm to estimate stiffness during multi-passes tooling tasks. The novelty of our approach is that the perturbations (required to estimate stiffness) originate from the intrinsic variability of the task itself rather than from a dedicated system as in all previous works which, specifically for this reason, are not easily applicable to real-life scenarios.

The task is a simplified version of an actual tooling task. Although the forearm is vertically supported to reduce fatigue, see Figure 5, the wrist is free to move so that RUD motions of the wrist induce a vertical motion of the tool, while FE motions of the wrist induce a horizontal motion of the tool. The operator is required to perform a series of FE motions (bottom plot in Figure 6) while maintaining a constant vertical force. Relatively ample motions (≈±0.1 rad) along the FE axis induce much smaller still significant (for the purpose of estimating RUD stiffness) motions along the RUD (≈±0.03 rad, as in Figure 8) as well as subsequent variations of the RUD torque, ultimately responsible for the vertical force imposed during the tooling task. Our regression detects exactly this variability, which occurs at a very low frequency (it takes more than one second for a full FE sweep, see bottom plot in Figure 6), i.e., we estimate quasi-static stiffness.

For validation purposes, the same method was used with an impedance-controlled robot used to carry out a similar task and verify that our data analysis and regressions could correctly predict the pre-programmed robot impedance. We were able to accurately predict the pre-programmed robot impedance providing confidence that the estimated values impedance parameters are correct when measuring human operators. The electromyographic activation correlated positively with the four different levels of exerted force (or RU torque), as expected from physiology.

Our method is meant to provide a practical approach to estimate human stiffness during tooling tasks for the purpose of programming a robot by demonstration. A clear limitation of our work is that we assumed a constant impedance for the human throughout the motion. This is clearly an oversimplification, partly justified by a relatively constrained range of flexion-extension motion (±45 deg for the FE axis) and the relatively constrained exerted forces (3–9 N). One possibility to overcome this limitation is to ensure repetitive motions and regress impedance at different postures by averaging along ensembles, rather than along time, as recently suggested by Ludvig and Perreault [34]. However, in many applications the robot impedance would require a single value input, therefore we feel that our approximation is acceptable.

Another limitation of our work is that we only focus on the RU joint, for which a constant angle and torque was maintained throughout the task. While the intended periodic motion along the FE axis (for this task) for robots is pre-programmed and therefore known, humans cannot easily estimate it. Currently, we only focused on the stationary axis (RU, for the given task) but continue to investigate the possibility to filter out the voluntary, self-paced motion. If successful, this will allow extending the stiffness identification for other motions.
